# Hemodialysis Nonattendance: Patient Characteristics and Outcomes in a Single Renal Center in North West England

**DOI:** 10.1111/hdi.13227

**Published:** 2025-03-06

**Authors:** Rajkumar Chinnadurai, Jessica Dean, Sharmilee Rengarajan, Julie Gorton, Ivona Baricevic‐Jones, Philip A. Kalra, Dimitrios Poulikakos

**Affiliations:** ^1^ Salford Royal Hospital, Northern Care Alliance NHS Foundation Trust Salford UK; ^2^ Faculty of Biology, Medicine and Health University of Manchester Manchester UK

**Keywords:** all‐cause mortality, dialysis nonattendance, hemodialysis, hospitalization, mental health

## Abstract

**Introduction:**

Nonattendance for prescribed hemodialysis (HD) sessions is a form of nonadherence that compromises the delivery of life‐sustaining HD therapy and is associated with severe morbidity and mortality. In this study, we aimed to assess the characteristics and outcomes of HD nonattenders in a single renal center in the North West of England.

**Methods:**

HD patients followed by the renal team at our unit between December 2020 and September 2022 were included in this study. Dialysis nonattendance data were retrieved from the incident reports (DATIX) between December 2020 and November 2022, excluding dialysis nonattendance due to concurrent hospitalization. The cohort was split into group 1: no dialysis nonattendance; group 2: two or fewer dialysis nonattendances; and group 3: more than two dialysis nonattendances for comparative analysis. All patients were followed up for outcomes including all‐cause mortality, transplantation, and hospitalizations until the study endpoint date of 12/31/2023. Predictors of dialysis nonattendance were identified using logistic regression.

**Results:**

Of the 464 patients, dialysis nonattendance was noted in 149 (32%) patients, of which 79 (17%) had two dialysis nonattendance episodes and 70 (15%) had more than two dialysis nonattendance episodes. Over a median follow‐up of 35 months, patients in group 3 had a higher hospitalization episode (4 vs. 1 day, *p* < 0.001) and lower kidney transplantation rates (4.3% vs. 13%, *p* = 0.038) compared to patients in group 1. In multivariate regression analysis, younger age (OR: 0.97; 95% CI: 0.95–0.98; *p* = 0.001), history of smoking (OR: 2.01; 95% CI: 1.12–3.62; *p* = 0.019), alcohol excess history (OR: 3.49; 95% CI: 1.87–6.49; *p* < 0.001) and history of mental health illness (OR: 3.01; 95% CI: 1.61–5.62; *p* = 0.001) were significant predictors of dialysis nonattendance.

**Conclusion:**

Skipping HD is a common phenomenon associated with mental health issues and is associated with increased morbidity. Further research is required to understand the psychosocial determinants of nonadherence and effective models of intervention developed to improve outcomes.

## 1 Introduction

Hemodialysis (HD) is a life‐sustaining renal replacement therapy option for patients with end‐stage kidney disease. The UK Kidney Association guidelines recommend maintenance HD to be a minimum of thrice weekly for a duration of four hours for each treatment. Nonadherence to prescribed HD treatment, including missing HD sessions or reducing session duration or nonadherence to dietary (low phosphate/potassium) or fluid restriction recommendations, is shown to be associated with increased mortality and hospitalization rates [1, 2, 3]. The estimated prevalence of patients missing more than one session of HD per month over a period of 4 months varies across different countries, from as low as 0.2% in Japan, 2.6% in the UK, and as high as 12.2% in the US, in data from the Dialysis Outcomes and Practice Patterns Study (DOPPS) [4].

Despite the often life‐threatening or fatal consequences of missed dialysis sessions [5], the high financial cost incurred by resulting emergency admissions [3], and additional requirements for HD treatment slots, this phenomenon of dialysis nonattendance remains poorly understood, and evidence‐based interventions for this cohort of patients are lacking.

While it is known that socioeconomic and psychosocial factors determine adherence to therapy in chronic kidney disease patients [6] and dialysis patients [7], there is limited research focused on dialysis nonattendance, which represents the extreme end of the spectrum of nonadherence.

To this end, we aimed to investigate the characteristics, risk factors, and outcomes for dialysis nonattendance at our center. We also explored the engagement of this group of patients with the psychological support offered.

## 2 Methods

### 2.1 Study Cohort Selection

**FIGURE 1:**
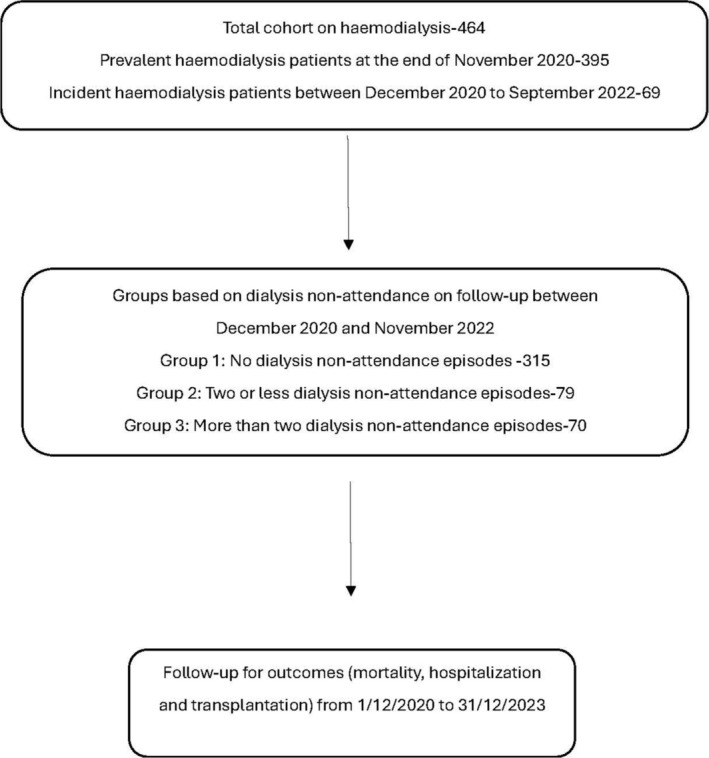
Flowchart of patient selection to the study.

Patient selection for this single‐center retrospective observational study was performed on a cohort of 464 (395 prevalent and 69 incident) in‐center HD patients followed by the renal team at the Northern Care Alliance NHS Foundation Trust between December 2020 and September 2022. A dialysis nonattendance list, which included 149 patients, was obtained from the incident reports (DATIX) generated every time a patient did not attend a dialysis session between December 2020 and November 2022. The dialysis nonattendance list excluded nonattendance due to concurrent hospitalizations. A comparative analysis was conducted between patients with no dialysis nonattendance (group 1), one or two dialysis nonattendance episodes (group 2) and those greater than two dialysis nonattendance episodes (group 3). The baseline date was taken as 12/1/2020 for prevalent dialysis patients and the date of the start of dialysis for incident patients. All patients were followed up for outcomes (all‐cause mortality, transplantation, and hospitalizations) until the occurrence of endpoints, including death, change of modality or transplantation, stopping of dialysis, and the study end date 12/31/2023. A flowchart of patient selection for the study is shown in Figure 1. To also enable comparison to the DOPPS of nonattendance, a separate analysis was conducted based on dialysis nonattendance in the first 4 months of the study by stratifying the cohort into two groups (no dialysis nonattendance and one or more dialysis nonattendance) as described by Salmi I et al. [4] The study baseline date and endpoints were kept similar to the total analysis.

### 2.2 Data Collection and Study Definitions

Data including demographics, comorbidities (diabetes mellitus, hypertension, and cardiovascular disease), smoking history, alcohol excess history, history of mental health illness, marital status, dialysis access, new referral to psychology, psychology clinic nonattendance, transplantation, mortality, and hospitalization episodes/dates were collected from electronic patient records (EPR). Patients are routinely assessed by the dialysis nurses and clinically reviewed by the consultants during bimonthly HD reviews. If any psychosocial issues are identified, the patients are referred to specialist renal psychology services.

Ethnicity and marital status (married or single) were obtained as recorded in demographic data. A history of smoking was defined as a current or past history of smoking, and an alcohol history was defined as alcohol intake above the UK recommended limits (> 14 units of alcohol per week). A history of a mental health condition (anxiety, depression, schizophrenia, etc.) as recorded in clinical records or intake of antidepressant medications apart from the management of pain (citalopram, sertraline, etc.) defined history of mental health illness. A cardiovascular history was defined as a history of ischemic heart disease, myocardial infarctions, cerebrovascular accident, congestive cardiac failure, and peripheral vascular disease. The indices of multiple deprivation (IMD) deciles were generated using the postcode of residence in the English Indices of Deprivation 2019 online portal (https://imd‐by‐postcode.opendatacommunities.org/imd/2019). The neighboring decile scores were grouped to achieve quintiles. Hospitalization episodes included admissions for acute illness and excluded elective hospital admissions for vascular access interventions such as arteriovenous fistula angioplasty or dialysis line removal. Data on psychology appointments and nonattendance or cancelations of these appointments were collected from EPR.

### 2.3 Statistical Analysis

In the descriptive part of the analysis, continuous variables were expressed as median and interquartile range, and the Mann–Whitney *U* test was used to generate the *p*‐value of the difference between the groups. Categorical variables were expressed as numbers and percentages, with *p*‐values obtained by the Chi‐square test. Univariate and multivariate binary logistic regression analyses assessed the predictors of dialysis nonattendance, with factors that were significant in the univariate model included in the multivariate model. A Kaplan–Meier chart was used to demonstrate the survival probability between the groups. A *p* value of < 0.05 was taken as statistical significance throughout the analysis. All analyses were conducted using SPSS version 26.

## 3 Results

### 3.1 Characteristics and Outcomes on Follow‐Up of the Total Cohort

Baseline characteristics and outcomes are presented in Table 1.

**TABLE 1:** Baseline characteristics and outcome of the cohort based on dialysis nonattendance groups.

Characteristics	Total: 464	Group 1: no nonattendance episodes, 315 (68%)	Group 2: one or two nonattendance episodes, 79 (17%)	Group 3: more than two nonattendance episodes, 70 (15%)	*p* between group 1 versus group 3
Age, years	63 (51–73)	64 (55–74)	62 (50–68)	55 (41–68)	< 0.001
Sex, male	289 (62.3%)	202 (64.1%)	44 (55.7%)	43 (61.4%)	0.671
Ethnicity, white	331 (71.3%)	209 (66.3%)	61 (77.2%)	61 (87.1%)	0.001
Lower IMD quintiles (1–3)	410 (88.4%)	274 (87%)	70 (88.6%)	66 (94.3%)	0.085
Smoking history	130 (28%)	74 (23.5%)	20 (25.35)	36 (51.4%)	< 0.001
Alcohol history	85 (18.3%)	39 (12.4%)	17 (21.5%)	29 (41.4%)	< 0.001
Mental health illness	84 (18.2%)	44 (14.1%)	14 (1.7%)	26 (37.1%)	< 0.001
Marital status, single	206 (44.4%)	146 (46.3%)	38 (48.1%)	22 (31.4%)	0.058
Diabetes mellitus	226 (48.7%)	155 (49.2%)	43 (54.4%)	28 (40%)	0.163
Hypertension	355 (76.5%)	231 (73.3%)	70 (88.6%)	54 (77.1%)	0.511
Cardiovascular disease	166 (35.8%)	105 (33.3%)	433 (41.8%)	28 (40%)	0.289
Dialysis access, AV fistula	297 (64.1%)	212 (67.5%)	46 (58.2%)	39 (55.7%)	0.143
Dialysis vintage, months	40 (22–65)	41 (22–70)	42 (26–61)	36 (24–61)	0.666
Outcomes					
Follow‐up, months	35 (14–37.5)	36 (14–37.5)	26 (13–37.5)	29 (15–37)	0.099
All‐cause mortality	175 (37.7%)	114 (36.2%)	30 (38%)	31 (44.3%)	0.206
New referral to psychology	81 (17.5%)	38 (12.1%)	16 (20.3%)	27 (38.6%)	< 0.001
Transplantation	53 (11.4%)	41 (13%)	9 (11.4%)	3 (4.3%)	0.038
Hospitalization episodes	2 (1–4)	1 (0–3)	2 (1–4)	4 (2–6)	< 0.001
Hospitalization days	12 (1–28)	8 (0–24)	13 (1–32)	20 (10–53)	< 0.001

*Note*: Continuous variables are expressed as median (interquartile range) and *p*‐value by the Mann–Whitney *U* test. Categorical variables are expressed as numbers (percentage) and *p*‐values by the Chi‐square test.

Abbreviations: AV, arteriovenous; IMD, index of multiple deprivation.

Of the 464 patients in the total cohort, dialysis nonattendance was noted in 149 (32%) patients, of whom 79 (17%) had two or fewer dialysis nonattendance episodes (group 2) and 70 (15%) had more than two dialysis nonattendance episodes (group 3). Patients in group 3 were significantly younger (55 vs. 64 years; *p* < 0.001), a higher proportion were of white ethnicity (87% vs. 66.3%; *p* = 0.001), had a history of smoking (51.4% vs. 23.5%; *p* < 0.001) and had a history of alcohol excess (41.4% vs. 12.4%; *p* < 0.001). A significantly higher proportion in group 3 had a history of mental health illness (37% vs. 14.1%; *p* < 0.001).

Over a median follow‐up of 35 months, patients in group 3, compared to group 1, had a lower kidney transplantation rate (4.3% vs. 13%; *p* = 0.038), a greater proportion referred to psychology services (39% vs. 12.1%; *p* < 0.001), higher median hospitalization episodes (4% vs. 1%; *p* < 0.001) and median hospitalization days (20 vs. 12 days; *p* < 0.001).

**FIGURE 2:**
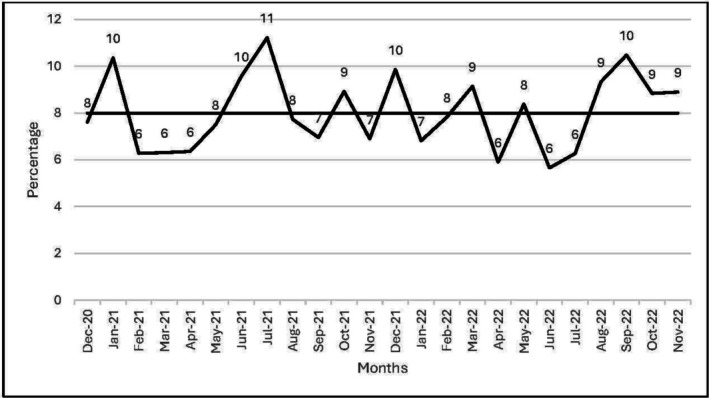
Monthly spread of unique patients’ dialysis nonattendance given as a percentage of the prevalent patients on dialysis.

In group 3, 94.3% of patients were from the lower three IMD quintiles, and the all‐cause mortality rate was 44.3% compared to 87% and 36.2%, respectively, in Group 1; however, these differences did not reach statistical significance (Table 1). A split of dialysis nonattendance based on age groups, ethnicity, and IMD quintiles is shown in Table S1. The average number of patients per month who did not attend dialysis as a percentage of the total prevalent cohort was noted to be 8% (Figure 2).

### 3.2 Predictors of Dialysis Nonattendance

**FIGURE 3:**
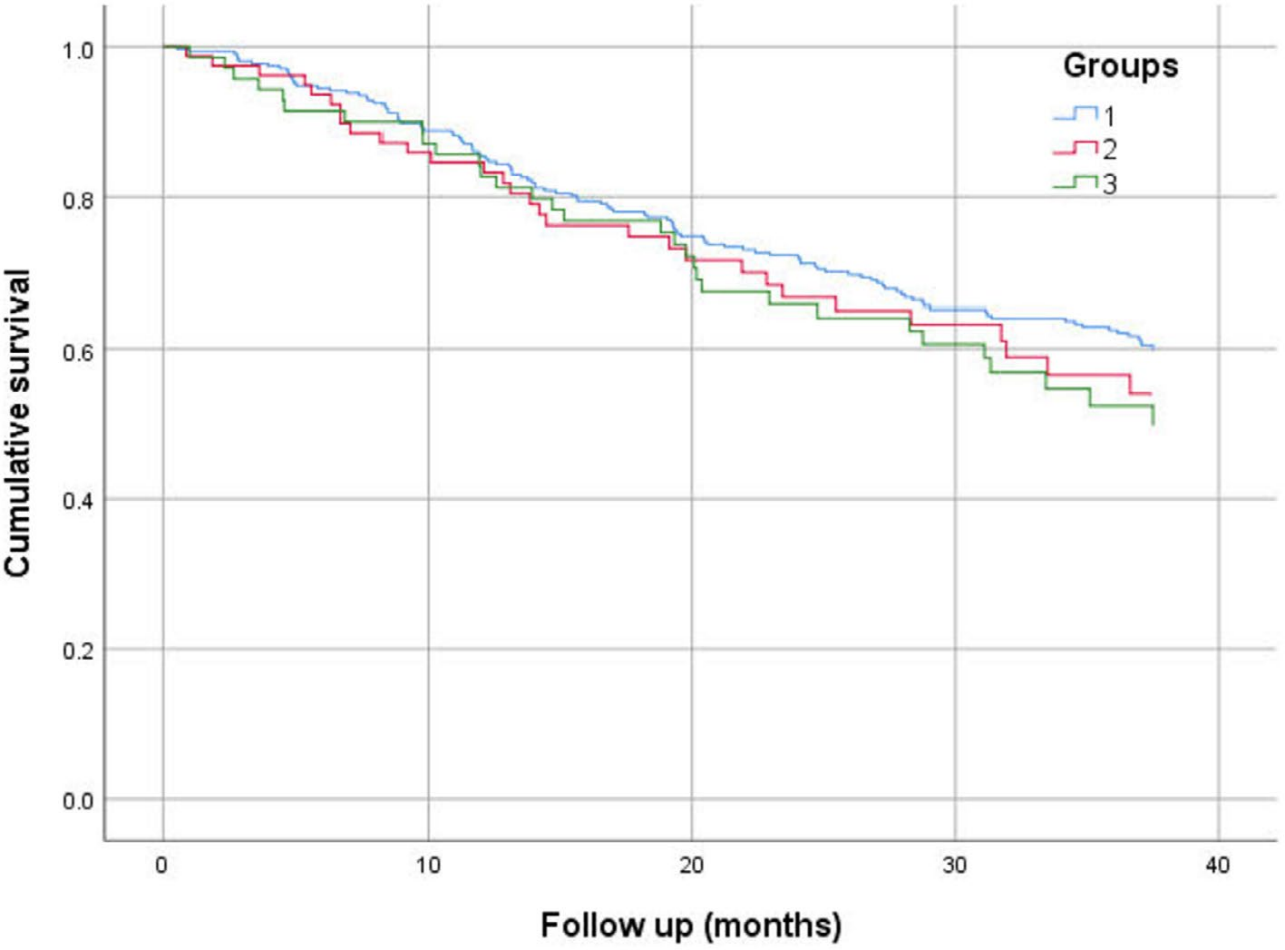
Kaplan–Meier chart for all‐cause mortality based on dialysis nonattendance groups.

A univariate logistic regression model looking at predictors of dialysis nonattendance (more than two episodes) showed that younger age, white ethnic background, history of smoking, alcohol excess, mental health illness, and being single in marital status are risk factors. A multivariate model showed younger age (OR: 0.97; 95% CI: 0.95–0.98; *p* = 0.001), history of smoking (OR: 2.01; 95% CI: 1.12–3.62; *p* = 0.019), alcohol excess history (OR: 3.49; 95% CI: 1.87–6.49; *p* < 0.001), and history of mental health illness (OR: 3.01; 95% CI: 1.61–5.62; *p* = 0.001) were significant independent predictors of dialysis nonattendance (Table 2). The Kaplan–Meier curve did not show a significant survival difference between the no dialysis nonattendance and dialysis nonattendance groups (Log‐rank, *p* = 0.378) (Figure 3).

**TABLE 2:** Predictors of dialysis nonattendance (more than two episodes) by binary logistic regression analysis (464 patients).

Variables	Univariate model		Multivariate model 1	
	OR (95% CI)	*p*	OR (95% CI)	*p*
Age	0.97 (0.95–0.98)	< 0.001	0.97 (0.95–0.98)	0.001
Gender, male	0.98 (0.58–1.67)	0.95		
Ethnicity, white	3.17 (1.53–6.59)	0.002	2.17 (0.98–4.79)	0.055
Lower IMD quintiles (1‐ 3)	1.88 (0.72–4.89)	0.196		
Smoking history	3.51 (2.08–5.91)	< 0.001	2.01 (1.12–3.62)	0.019
Alcohol excess history	4.49 (2.59–7.79)	< 0.001	3.49 (1.87–6.49)	< 0.001
Mental health illness	3.31 (1.89–5.79)	< 0.001	3.01 (1.61–5.62)	0.001
Marital status, single	1.81 (1.06–3.11)	0.028	1.61 (0.89–2.91)	0.110
Hypertension	1.06 (0.58–1.94)	0.836		
Diabetes mellitus	0.64 (0.38–1.07)	0.091		
Cardiovascular disease	1.29 (0.77–2.16)	0.334		
Dialysis access, AV fistula	0.62 (0.37–1.03)	0.067		
Dialysis vintage, months	0.99 (0.98–1.00)	0.064		

*Note*: Multivariate model included variables that were significant in the univariate model.

Abbreviations: AV, arteriovenous; CI, confidence interval; IMD, index of multiple deprivation; OD, odds ratio.

### 3.3 Subgroup Analysis Based on DOPPS Comparison (353 Patients)

A further subgroup analysis based on DOPPS comparison categories included 315 patients with no dialysis nonattendance episodes and 38 (10.8%) patients who had one or more dialysis nonattendance episodes in the first four months of follow‐up. The analysis showed similar results to those of the entire follow‐up period, with patients in the dialysis nonattendance group being younger (57 vs. 64 years, *p* = 0.023), a higher proportion from a white ethnic background (87% vs. 66%, *p* = 0.002) and having a history of mental health illness (37% vs. 14%, *p* < 0.001). Dialysis vintage was noted to be shorter in patients who missed dialysis in the first 4 months (20 vs. 41 months, *p* < 0.001). Patients in the dialysis nonattendance group also had poor outcomes, including higher hospitalization episodes (3 vs. 1; *p* = 0.002) and higher median hospitalization days (20 vs. 8 days, *p* = 0.002) (Table S2).

### 3.4 Psychology Clinic Referrals and Nonattendance

**FIGURE 4:**
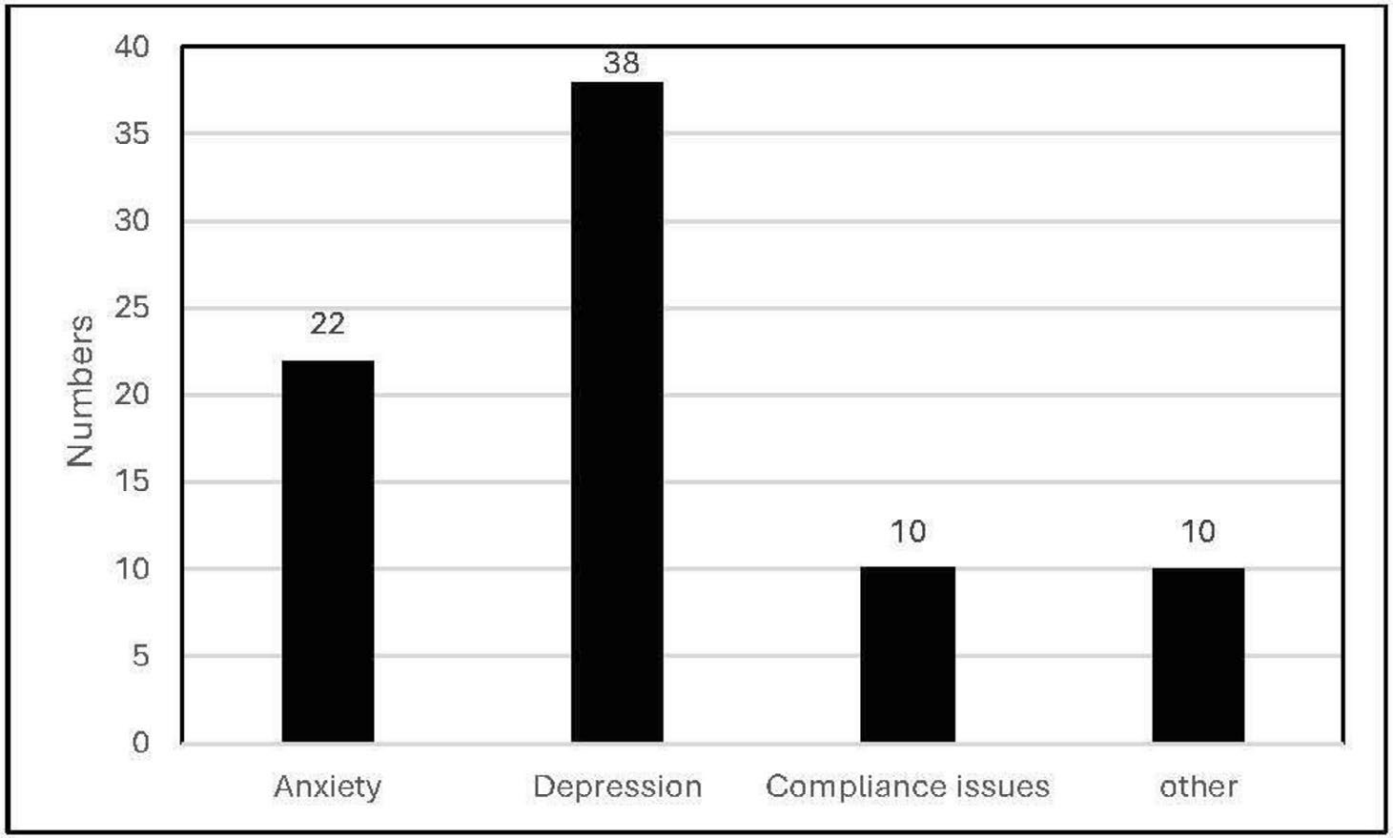
Reasons for referral to psychology services (total‐81 referrals).

During the follow‐up period, a total of 81 patients were referred to the psychology services, with chief reasons being anxiety (27%), depression (47%), and adherence issues (12.4%) (Figure 4). Of the total of 603 psychology appointments offered, 211 (35%) were not attended or canceled, with 122 (58%) of these relating to patients in the dialysis nonattendance group. There were no statistically significant differences between the characteristics of the patients who did or did not attend or cancel their appointment (Table S3).

## 4 Discussion

This study shows that recurrent dialysis nonattendance is quite common in our HD population, with 15% of patients having more than two dialysis nonattendance episodes during an average follow‐up of just under 3 years. Dialysis nonattendance was associated with increased mortality, hospitalization, and lower transplant rates. In our cohort, younger age, a white ethnic background, a history of high‐risk behaviors such as smoking, alcohol excess, and mental health illness were significantly associated with frequent dialysis nonattendance.

Our results align with previous studies reporting an independent association between younger age [8, 9, 10] and missed dialysis treatment. It has been speculated that younger individuals may underestimate the risk of missing treatment due to self‐perception of healthier physical status compared to most of their peers [11], however, additional psychological factors may contribute to this behavior. In a study using patients’ self‐assessed physical and mental functional health status, DeOreo et al. [12]. reported that patients who did not attend for more than two HD sessions per month had higher physical and lower mental health status scores as judged by themselves.

Interestingly, in our study, we also found an association between patients from White ethnic backgrounds and increased risk of recurrent dialysis nonattendance and psychology referrals. In a previous US study [2], Black ethnic backgrounds were more likely to skip dialysis sessions compared to whites. In the UK, the white population is more likely to have been diagnosed with mental health illness, which is also more prevalent in the lowest quintiles of IMD [13]. The ethnic minority population in our study consisted predominantly of South Asians, representing the ethnic demographic of our catchment population. It could be speculated that family or cultural factors may have played a protective role. Further, a stigma of mental health illness in these communities can be a factor limiting reporting psychological issues. A similar paradoxical ethnic association with adverse outcomes has been reported in Asian children in the UK being less likely to be under child protection services despite higher poverty rates compared to White children [14] and in Latino children in the US, the so‐called “Hispanic paradox” where the relative risk of referral to child services was significantly lower compared to White children [15].

In our study, 94.3% of patients with more than two episodes of dialysis non‐attendance were from the lowest quintile of the IMD compared to 87% of the remaining population. The required sample size to detect statistical significance (alpha 0.05, power 80%) would be 195; however, our sample size of > 2 dialysis nonattendances involved only 70 patients and therefore could not provide adequate power for comparative analysis. Furthermore, the IMD is a relative measure of deprivation based on geographical areas and does not provide socioeconomic data on an individual level or information related to the level of access to social support (benefits, etc.) in eligible patients, which may be suboptimal in patients who exhibit nonadherence.

Our finding that diagnosed mental health illness or new need for psychological input was significantly associated with increased risk of dialysis nonattendance confirms reports from US studies [4, 9, 16] showing that a history of depressive symptoms is an independent risk factor for dialysis nonattendance. In our study approximately one third of the psychology outpatient appointments were canceled or not attended by patients. This finding indicates that traditional models for psychological support based on outpatient visits inevitably face the same challenge of nonadherence and underscores the need to investigate alternative multidisciplinary approaches with psychological interventions integrated within routine dialysis care and facilitated by the close relationship these patients develop with the immediate team that delivers dialysis every other day.

The subanalysis in our study using DOPPS methodology showed similar nonattendance rates, confirming a previous US report that the DOPPS sampling design produces population‐representative data [17]. In the subanalysis, although we confirmed an increased risk of hospitalization in the group with > 1 skipped HD session over the first 4 months, we did not detect a statistically significant difference in mortality, possibly due to limited sample size. Further, patients who missed their dialysis in the first 4 months had a shorter dialysis vintage, probably reflecting that patients develop a better understanding of the importance of dialysis once they are on it for some time or possibly due to a shorter follow‐up period.

Study limitations include the lack of social deprivation data at the individual level, and we also did not conduct qualitative/thematic research on the group of recurrent nonattenders to identify common patterns that could shed more light on psychological characteristics or patterns of beliefs and behaviors that could in turn inform effective interventions. In this study, we did not investigate associations with other measures of nonadherence (fluid, dietary, medication adherence, hesitancy to undergo arteriovenous fistula formation). In the seminal study by Port et al. [18]. the association between skipping dialysis treatment and mortality was seen to be in excess of a simple relative reduction in KT/V, indicating that other adverse patient behaviors are likely to coexist in patients nonattending for their dialysis treatment. Finally, in this retrospective study, we were not able to collect data on health literacy, a potentially modifiable factor that may play a role in health behaviors [19, 20]. Limited health literacy was noted to be significantly and independently associated with adverse clinical events, missed dialysis sessions, and mortality [21].

In conclusion, recurrent dialysis nonattendance is a common problem associated with mental health issues that confers a high risk that is not addressed using traditional models of intervention. Further research is required to understand the psychosocial characteristics of nonattenders and to design effective interventions to improve adherence.

## Conflicts of Interest

The authors declare no conflicts of interest.

## Supporting information


Data S1.

## Data Availability

The data that support the findings of this study are available from the corresponding author upon reasonable request.

## References

[1] R. Saran , J. L. Bragg‐Gresham , H. C. Rayner , et al., “Nonadherence in Hemodialysis: Associations With Mortality, Hospitalization, and Practice Patterns in the DOPPS,” Kidney International 64, no. 1 (2003): 254–262. 12787417 10.1046/j.1523-1755.2003.00064.x

[2] J. E. Leggat, Jr. , S. M. Orzol , T. E. Hulbert‐Shearon , et al., “Noncompliance in Hemodialysis: Predictors and Survival Analysis,” American Journal of Kidney Diseases 32, no. 1 (1998): 139–145, 10.1053/ajkd.1998.v32.pm9669435. 9669435

[3] J. Fotheringham , M. T. Smith , M. Froissart , et al., “Hospitalization and Mortality Following Non‐Attendance for Hemodialysis According to Dialysis Day of the Week: A European Cohort Study,” BMC Nephrology 21, no. 1 (2020): 218. 32517695 10.1186/s12882-020-01874-x PMC7285433

[4] A. Salmi, I , M. Larkina , M. Wang , et al., “Missed Hemodialysis Treatments: International Variation, Predictors, and Outcomes in the Dialysis Outcomes and Practice Patterns Study (DOPPS),” American Journal of Kidney Diseases 72, no. 5 (2018): 634–643. 30146421 10.1053/j.ajkd.2018.04.019

[5] J. A. Parsons , D. M. Taylor , F. J. Caskey , and J. Ives , “Ethical Duties of Nephrologists: When Patients Are Nonadherent to Treatment,” Seminars in Nephrology 41, no. 3 (2021): 262–271. 34330366 10.1016/j.semnephrol.2021.05.007

[6] W. Tesfaye , N. Parrish , K. Sud , A. Grandinetti , and R. Castelino , “Medication Adherence Among Patients With Kidney Disease: An Umbrella Review,” Advances in Kidney Disease and Health 31, no. 1 (2024): 68–83. 38403396 10.1053/j.akdh.2023.08.003

[7] S. Clark , K. Farrington , and J. Chilcot , “Nonadherence in Dialysis Patients: Prevalence, Measurement, Outcome, and Psychological Determinants,” Seminars in Dialysis 27, no. 1 (2014): 42–49. 24164416 10.1111/sdi.12159

[8] A. J. Bleyer , B. Hylander , H. Sudo , et al., “An International Study of Patient Compliance With Hemodialysis,” Journal of the American Medical Association 281, no. 13 (1999): 1211–1213. 10199431 10.1001/jama.281.13.1211

[9] K. E. Chan , R. I. Thadhani , and F. W. Maddux , “Adherence Barriers to Chronic Dialysis in the United States,” J Am Soc Nephrol 25, no. 11 (2014): 2642–2648. 24762400 10.1681/ASN.2013111160 PMC4214530

[10] K. Cambell , A. Millard , G. McCartney , S. McCullough , and NHS Scottland , “Who is Least Likely to Attend? An Analysis of Outpatient Appointment DNA Data in NHS Dumfries & Galloway,” (2015), https://www.scotpho.org.uk/media/1165/scotpho150319‐dna‐analysis‐nhs‐dumfries‐and‐galloway.pdf.

[11] N. G. Kutner , R. Zhang , W. M. McClellan , and S. A. Cole , “Psychosocial Predictors of Non‐Compliance in Haemodialysis and Peritoneal Dialysis Patients,” Nephrology, Dialysis, Transplantation 17, no. 1 (2002): 93–99. 10.1093/ndt/17.1.93 11773470

[12] P. B. DeOreo , “Hemodialysis Patient‐Assessed Functional Health Status Predicts Continued Survival, Hospitalization, and Dialysis‐Attendance Compliance,” American Journal of Kidney Diseases 30, no. 2 (1997): 204–212, 10.1016/s0272-6386(97)90053-6. 9261030

[13] T. Watt , A. Raymond , and L. Rachet‐Jacquet , “Quantifying Health Inequalities in England, the Health Foundation,” (2022), https://www.health.org.uk/news‐and‐comment/charts‐and‐infographics/quantifying‐health‐inequalities.

[14] P. Bywaters , J. Kwhali , G. Brady , T. Sparks , and E. Bos , “Out of Sight, out of Mind: Ethnic Inequalities in Child Protection and Out‐Of‐Home Care Intervention Rates,” British Journal of Social Work 47, no. 7 (2017): 1884–1902.

[15] E. Putnam‐Hornstein , B. Needell , B. King , and M. Johnson‐Motoyama , “Racial and Ethnic Disparities: A Population‐Based Examination of Risk Factors for Involvement With Child Protective Services,” Child Abuse & Neglect 37, no. 1 (2013): 33–46. 23317921 10.1016/j.chiabu.2012.08.005

[16] S. D. Weisbord , M. K. Mor , M. A. Sevick , et al., “Associations of Depressive Symptoms and Pain With Dialysis Adherence, Health Resource Utilization, and Mortality in Patients Receiving Chronic Hemodialysis,” Clinical Journal of the American Society of Nephrology 9, no. 9 (2014): 1594–1602. 25081360 10.2215/CJN.00220114 PMC4152801

[17] B. Robinson , D. Fuller , D. Zinsser , et al., “The Dialysis Outcomes and Practice Patterns Study (DOPPS) Practice Monitor: Rationale and Methods for an Initiative to Monitor the New US Bundled Dialysis Payment System,” American Journal of Kidney Diseases 57, no. 6 (2011): 822–831. 21530036 10.1053/j.ajkd.2011.03.001 PMC3885981

[18] F. K. Port , R. A. Wolfe , D. C. Stannard , et al., “The Dose of Hemodialysis and Patient Mortality,” Kidney International 50, no. 2 (1996): 550–556, 10.1038/ki.1996.348. 8840285

[19] U. Elisabeth Stømer , A. Klopstad Wahl , L. Gunnar Gøransson , and K. Hjorthaug Urstad , “Health Literacy in Kidney Disease: Associations With Quality of Life and Adherence,” Journal of Renal Care 46, no. 2 (2020): 85–94. 31950601 10.1111/jorc.12314

[20] R. E. Billany , A. Thopte , S. F. Adenwalla , D. S. March , J. O. Burton , and M. P. M. Graham‐Brown , “Associations of Health Literacy With Self‐Management Behaviours and Health Outcomes in Chronic Kidney Disease: A Systematic Review,” Journal of Nephrology 36, no. 5 (2023): 1267–1281, 10.1007/s40620-022-01537-0. 36645651 PMC10333418

[21] D. M. Taylor , S. Fraser , C. Dudley , et al., “Health Literacy and Patient Outcomes in Chronic Kidney Disease: A Systematic Review,” Nephrology, Dialysis, Transplantation 33, no. 9 (2018): 1545–1558. 10.1093/ndt/gfx293 29165627

