# Supplementary material for: Hemodialysis Nonattendance: Patient Characteristics and Outcomes in a Single Renal Center in North West England
Source: Hemodial Int. 2025 Mar 6;29(3):363–70. doi: 10.1111/hdi.13227 (PMC12287893; doi:10.1111/hdi.13227)
Supplement: Supplementary file 1 — Data S1. [file HDI-29-363-s001.docx]

**Supplementary table 1. Age group, Ethnicity and IMD quintiles based on dialysis non-attendance**

| Characteristics | Group 1  No non-attendance  episodes  315 | Group 2  One or two non-attendance  episodes  79 | Group 3  More than two non-attendance  episodes  70 | p-Value between  groups |
| --- | --- | --- | --- | --- |
| Age group <30  31-40  41-70  >70 | 13 (4.1%)  16 (5.1%)  184 (58.4%)  102 (32.4%) | 4 (5.1%)  9 (11.4%)  50 (63.3%)  16 (20.3%) | 6 (8.6%)  11 (15.7%)  43 (61.4%)  10 (14.3%) | **0.002** |
| Ethnicity, White  Asian  African  Other | 209 (66.3%)  87 (27.6%)  13 (4.1%)  6 (1.9%) | 61 (77.2%)  10 (12.7%)  6 (7.6%)  2 (2.5%) | 61 (87.1%)  6 (8.6%)  2 (2.9%)  1 (1.4%) | **0.003** |
| IMD quintiles 1  2  3  4  5 | 181 (57.5%)  57 (18.1%)  36 (11.4%)  31 (9.8%)  10 (3.2%) | 53 (67.1%)  12 (15.2%)  5 (6.3%)  6 (7.6%)  3 (3.8%) | 48 (68.6%)  13 (18.6%)  5 (7.1%)  4 (5.7%)  0 | 0.518 |

IMD- index of multiple deprivation. Categorical variables are expressed as numbers (percentage) and p-values by the Chi-square test.

**Supplementary table 2. Comparison of baseline characteristics and outcomes between no dialysis non-attendance and dialysis non-attendance in the first 4 months**

| **Variable** | **Total**  **353** | **No non-attendance**  **315** | **Non-attendance in first 4 months (>1)**  **38** | **p-value** |
| --- | --- | --- | --- | --- |
| Age | 64 (53-74) | 64 (55-74) | 57 (42-69) | **0.023** |
| Gender, male | 228 (64.6%) | 202 (64.1%) | 26 (68.4%) | 0.601 |
| Ethnicity, White | 242 (68.6%) | 209 (66.3%) | 33 (86.8%) | **0.010** |
| Lower IMD quintiles (1- 3) | 309 (87%) | 274 (87%) | 35 (92.1%) | 0.367 |
| Smoking history | 94 (26.6%) | 74 (23.5%) | 20 (52.6%) | **<0.001** |
| Alcohol excess history | 50 (14.2%) | 39 (12.4%) | 11 (28.9%) | **0.006** |
| Mental health illness | 58 (16.5%) | 44 (14.1%) | 14 (36.8%) | **<0.001** |
| Marital status, single | 190 (53.8%) | 169 (53.7%) | 21 (55.3%) | 0.851 |
| Hypertension | 262 (74.2%) | 231 (73.3%) | 31 (81.6%) | 0.272 |
| Diabetes Mellitus | 176 (49.9%) | 155 (49.25) | 21 (55.3%) | 0.481 |
| Cardiovascular disease | 124 (35.1%) | 105 (33.3%) | 19 (50%) | **0.042** |
| Dialysis access, AV fistula | 231 (65.6%) | 212 (67.5%) | 19 (50%) | 0.088 |
| Dialysis Vintage, months | 38 (19.4-65) | 41 (22-70) | 20 (12-31.7) | **<0.001** |
| Outcome |  |  |  |  |
| Follow-up, months | 32 (13-37.5) | 36 (14-37.5) | 20 (12-31.6) | **<0.001** |
| Referred to psychology | 34 (13.6%) | 24 (11.3%) | 10 (26.3%) | **0.013** |
| All-cause mortality | 128 (36.3%) | 114 (36.2%) | 14 (36.8%) | 0.937 |
| Transplantation | 42 (11.9%) | 41 (13%) | 1 (2.6%) | 0.062 |
| Hospitalization episodes | 2 (0-3) | 1 (0-3) | 3 (1-5) | **0.002** |
| Hospitalization days | 10 (0-26) | 8 (0-24) | 20.5 (9-46) | **0.002** |

IMD- index of multiple deprivation, AV-arteriovenous, DNA-dialysis non-attendance

Continuous variables are expressed as median (interquartile range) and p-value by the Mann-Whitney U test. Categorical variables are expressed as numbers (percentage) and p-values by the Chi-square test.

**Supplementary table 3. Characteristics of patients who missed psychology appointments**

| Total -81 patients | Missed 2 or less appointments (48) | Missed more than 2 appointments (33) | p-value |
| --- | --- | --- | --- |
| Age, years | 51 (38-63) | 51 (38-59) | 0.744 |
| Sex, male | 26 (54.2%) | 13 (39.4%) | 0.191 |
| Ethnicity, White | 38 (79.2%) | 25 (87.9%) | 0.308 |
| Lower IMD Quintiles (1,2,3) | 43 (89.6%) | 31 (93.9%) | 0.493 |
| Smoking history | 21 (43.8%) | 8 (24.2%) | 0.072 |
| Alcohol history | 12 (25%) | 7 (21.2%) | 0.693 |
| Marital status, single | 29 (60.4%) | 19 (57.6%) | 0.798 |
| Dialysis non-attendance group | 24 (50%) | 19 (57%) | 0.502 |

IMD- index of multiple deprivation. Continuous variables are expressed as median (interquartile range) and p-value by the Mann-Whitney U test. Categorical variables are expressed as numbers (percentage) and p-values by the Chi-square test.
